# The Relation of Dentistry to Medicine

**Published:** 1882-05

**Authors:** W. W. Allport


### THE RELATION OF DENTISTRY TO MEDICINE.

BY DR. W. W. ALLPORT.

Delivered at the Banquet of the Michigan State Dental Association, March 31, 1882.

It is now twenty-seven years since I first passed through Michigan looking for a western home, which I found in the then young but promising city of Chicago.

Many times since that period various business has drawn me within the limits of your State. For several years I had interests at St. Joseph, which required my frequent attention, and I have often spent my summer vacations fishing in streams tributary to your upper lakes. My journeys to and from the East, which have not been few, have usually taken me through Detroit. In fact, so often has business and pleasure alike called me among you, and so much am I attached to your State, that I almost feel as though I should be reckoned one of her citizens, and that I ought to be a member of your Society, rather than your guest, to-night.

Our Union has few States more blest than yours in natural resources. It is rich in the products of agriculture, in native ores of copper and iron, in salines, and in vast tracts of forest, which contribute their wealth of building material to our treeless prairies, and add comfort and beauty to our homes. It is fruitful in enterprises and industries of all kinds, and it has a commerce of which many countries of the old world might well be proud; but its crowning glory is its educational institutions, from the district school to its splendid university, which ranks among the first, and in many respects, is first in our country.

And well would it have been had our other Western States so bountifully and wisely provided for the education of their sons and daughters. Its graduates are found in every State of the Union. From it has come some of our first scientists and most learned practitioners and teachers of law and medicine, as well as some of our most judicious law-makers in our State and national councils. To it is Chicago largely indebted for her most distinguished medical practitioners and teachers; and the recently established dental school in connection with its medical department, has been sending out graduates whose medical education is superior to that obtained in most of our regular dental colleges, and they are finding place and making for themselves position in our Western States.

And this brings me to the subject of the sentiment to which I am called upon to respond—"The Relation of Dentistry to Medicine."

To define this relation is not an easy task. The status of law, medicine, and divinity, as well as the various mechanical pursuits, are well defined, not only by those engaged in them, but by the public at large. But this is not the case with what is known as dentistry. Even those who practice it are not fully settled as to whether it is a mechanical pursuit, a profession by itself, or a department in medicine; and the facts in regard to its practice fully justify this undefined position. If I were asked whether dentistry was a mechanical business, my reply would be "yes." Or, if the inquiry were whether it constituted a special department in medical practice, I would still answer "yes." If asked whether it were a calling by itself, as many claim, I would also reply "yes"—according to its teaching and practice.

But how, you ask, can the same answer be given to these seemingly contradictory inquiries? To this my reply is, when a man practices what is popularly known as dentistry, such as removing tartar from the teeth, preparing cavities in teeth and filling them, and making sets of artificial teeth, no matter how well these operations may be performed mechanically, if he is ignorant of the cause of dental diseases and the medical science essential to their proper treatment, he is a mechanic and nothing more, as a large proportion are. In this case, so-called dentistry is in no way related to medicine. If, on the other hand, the practitioner is medically educated, is conversant with the cause of the diseases he is called upon to treat, and the relation they bear to associate parts in the manifold reflex action of the teeth upon other portions of the body, and the action of remote organs upon the teeth and their appendages, and if he brings the same intelligence to bear in the treatment of dental diseases that the ophthalomologist brings to bear upon the treatment of the eye, or the surgeon brings to bear upon the treatment of other surgical operations, which many do, then he is a special practitioner in medicine with all that the term implies. In *this* case, dentistry bears a most intimate relation to medicine.

But if he has sufficient mechanical skill to construct the simpler kinds of mechanical dentistry reasonably well, and possesses tolerable, or even marked skill in the mechanical manipulation of filling teeth, in what is known as operative dentistry, and has just enough medical knowledge not to be a physician, then I suppose it would be considered that he belonged to a distinct calling —our glorious profession.

The anomalous and strange position which our calling occupies at the present time will not be wondered at when we consider its history.

It is said, and generally with truth, that first impressions are the best, and when Dr. Harris was impressed with the idea that the diseases and treatment of the teeth should be taught in medical colleges, and that dental surgery should be made a specialty in medical practice, he was undoubtedly correct; and when a medical college refused to establish a chair for that purpose, future progress in dental science made the establishment of dental colleges a necessity. Hence, it was not from choice but necessity that our present system of dental teaching was inaugurated, and it is not impossible that the advance in the science and practice of dentistry has so far been quite as great under this system of teaching as it would have been had it at *first* been connected with medical colleges; and, under all the circumstances, we most certainly have just cause for congratulation that our dental colleges have done so much and so well what they have done, for there is no question that the proper practice of dentistry in its two departments has now become so complicated—the one with mechanics and art, and the other with mechanics and medical science—and the proper practice of each has become so intricate that there are few indeed who possess the natural gifts or could acquire the necessary and varied skill for the proper practice of both; and the further attempt of our dental colleges to teach both to the same individual, and to make a partial qualification in each essential to graduation, and the subsequent attempt on the part of these graduates to practice both departments only serves to dwarf both.

Proficiency of attainment in these two dissimilar vocations in the time now allotted to their study, is utterly impossible. When properly learned and practiced, one should be classed as a mechanical art, the other as a medical specialty.

The proper practice of dentistry as a distinct calling requires three leading and well-marked natural gifts:—mechanical talent, artistic feeling, and the ability to comprehend medical science—three qualities seldom found in the same individual. The first is mechanics; this is required in what is known as mechanical and as operative dentistry, and without which more than a moderate success in either would not be possible. But from this common ground their lines diverge and they have but little, if anything, in common. In the mechanical or prosthetic part of dentistry, mechanical talent should be supplemented by the same gifts which would either make a sculptor or a painter; and these gifts should be cultivated by art-studies rather than the medical studies essential for the dental surgeon.

Artificial dentures have three uses: mastication, aids to speech, and artistic or natural appearance in the mouth. Their fit and usefulness depends entirely upon a knowledge of the principles of mechanics and their correct application. A proper construction of artificial dentures so as to aid speech, involves a knowledge of anatomy and the action of the vocal organs, a lack of which knowledge will explain the reason that so many sets of teeth are impediments instead of aids to speech. Their natural appearance in the mouth depends entirely upon the application of principles of art, which are distinct from dental surgery.

Proficiency, therefore, in prosthetic dentistry requires study and skill far too great to be coupled with or tacked on to the tail of any department of medicine, the attempt to do which has rendered it simply as a mechanical business almost a disgrace; and I will venture the assertion that there is not at the present time, as generally practiced, a class of mechanical business so poorly followed, or that falls so far short of its possibilities as that known as mechanical dentistry. I will not except ready-made clothing or boots and shoes.

Now, we all know that for full sets of teeth there is nothing that will compare with what is known as continuous gum teeth, and of the 12,000 dentists in the United States, there are not 200 who can do this style of work properly, or even acceptably—and I doubt if there are 100. And there are not 1,000 who can creditably meet the various requirements of full and partial cases on gold plate. And yet, we all know that when these two kinds of work are properly done they are far superior, in most cases, to rubber or celluloid. Nor is this condition of things to be wondered at when we remember the skill that is required in putting up this better class of work, and the time and attention that is given to learn it.

Why, Mr. President, a man cannot learn to put up good gold or continuous gum work, even mechanically, to say nothing of its art, in less than two years, and he may do nothing else. But when is added to the mechanical part the selection and arrangement of teeth in the mouth to give a natural appearance, though he may possess an artistic gift, three years is as little time as should be devoted to learning it; and it is utterly impossible for any considerable number to learn the details and acquire skill in mechanical dentistry, and at the same time gain such a knowledge of medicine and acquire such skill in the treatment of dental diseases as will make them proper practitioners of dental surgery in the time usually devoted to it in the accepted mode of teaching.

For the credit of ourselves, as well as for the best interests of the public, this way of things should cease. But it never will so long as it is taught in its present manner.

There is enough in it to engage the entire time and attention of the finest mechanical and artistic talent, and it will never be elevated to its true position until it is learned and practiced as a distinct mechanical art uncomplicated with medicine.

On the other hand, the proper treatment of dental diseases requires a knowledge of medicine equal to the general practitioner, to which should be added such special knowledge as would make the dental surgeon an authority worthy to be taken into consultation with medical men in all diseases, either directly or remotely connected with the teeth; which would, of course, include sufficient knowledge to prescribe for all constitutional or local conditions that affect these organs. To this should be added such training as would make him a skilled manipulator in operations upon the teeth, or associate parts. To obtain such knowledge and acquire such skill, even to a reasonable degree, in the time now allotted by our dental colleges and at the same time to become respectable manipulators in the various kinds of mechanical dentistry, is simply impossible. And it will never be elevated to its proper position as a special department in medical practice until its teaching and practice is uncomplicated with mechanical dentistry.

With this statement, the practical question that confronts us is what change should be made in dental teaching to meet this exigency? Possibly this problem is not easily solved, but I will state what seems to me to be desirable. In the first place, I would require all dental surgeons to be medically educated; and I would have physicians so educated as to dental diseases that they would be as intelligent upon these matters as they are in regard to other diseases. To this end I would establish chairs upon diseases of the teeth in all medical colleges, and all entering students should matriculate as medical students, thus making a knowledge of the diseases of the teeth and the science of their treatment as essential to graduation as a knowledge in any other department of medicine—graduating upon the same footing. For the practical teaching of mechanical dentistry, as well as the practical application of medical science in the treatment of dental diseases, I would have an infirmary. In this infirmary I would have a department for the teaching of mechanical or prosthetic dentistry under the direction of one or more competent mechanical dentists, who would give instructions in all that related to that practice.

The students in this department should be required to attend such lectures as would make them familiar with the anatomy of the parts about which they work, as well as such lectures upon chemistry and art as would be applicable in their practice, and they should receive certificates of qualification on becoming sufficiently skilled in their calling to engage in the practice of mechanical dentistry only. In this infirmary there should be a department for didactic and clinical instruction for those proposing to become dental surgeons; which department, of course, should be under the direction of the most competent dental surgeons in the country—as clinical surgery is taught by the most experienced teachers and practitioners of surgery, rather than by young and inexperienced dentists, as is now too often the case in our dental colleges. But in no case should they receive certificates of qualification to practice without a medical degree. For the present, or until the people had become educated to the idea that mechanical dentistry could be more properly done by those who follow it as an exclusive business, it might be well to make it optional for those who propose to practice dental surgery to receive instruction in mechanical dentistry; and to give them a certificate of qualification for exactly what they were competent to do.

If they were competent to put up work on gold or platina, let it be so stated; and if they were only competent to put up work on rubber and celluloid, make a statement in exact accordance with these facts. But in no way make the qualification to practice in mechanical dentistry requisite to a certificate to practice in dental and oral surgery.

In this way mechanical dentistry would be elevated to a position of great usefulness and respectability. But it should not, nor could not, in any way be regarded as a department in medicine, or in any way related to its practice, any more than is the making of artificial limbs, or sculpture, or painting.

In this way, I should in a few years expect to see our practice permanently divided, and prosthetic dentistry developed into a high mechanical and artistic calling, and the relation between dental surgery and the other specialties, as well as the general practice of medicine, grow into a more proper and intimate relation.

In this way only, Mr. President, would the natural relation between dental surgery and medicine and mechanical dentistry and its kindred arts be developed and maintained; and it is to be hoped that the time is not far distant when the Regents of the University of Michigan will see fit to inaugurate this or some similar system of teaching. For we want no more eye doctors, tooth doctors or bone setters, but physicians, to treat the diseases of God's temple—the human organism.

